# Reinforced 3D Composite Structures of γ-, α-Al_2_O_3_ with Carbon Nanotubes and Reduced GO Ribbons Printed from Boehmite Gels

**DOI:** 10.3390/ma14092111

**Published:** 2021-04-22

**Authors:** Cristina Ramírez, Manuel Belmonte, Pilar Miranzo, Maria Isabel Osendi

**Affiliations:** Instituto de Cerámica y Vidrio (ICV), Consejo Superior de Investigaciones Científicas, CSIC, Kelsen 5, Cantoblanco, 28049 Madrid, Spain; mbelmonte@icv.csic.es (M.B.); pmiranzo@icv.csic.es (P.M.); miosendi@icv.csic.es (M.I.O.)

**Keywords:** reinforced 3D-printed ceramic, nanofiber reinforced 3D-printed materials, alumina graphene composites, ceramic graphene composites, graphene nanoribbons, multiwall carbon nanotubes, boehmite gels, γ-Al_2_O_3_, α-Al_2_O_3_

## Abstract

The ability of boehmite to form printable inks has sparked interest in the manufacturing of 3D alumina (Al_2_O_3_) and composite structures by enabling direct ink writing methods while avoiding the use of printing additives. These materials may exhibit high porosity due to the printing and sintering procedures, depending on the intended application. The 3D-printed porous composite structures of γ-Al_2_O_3_ and α-Al_2_O_3_ containing 2 wt.% of carbon nanotubes or reduced graphene oxide ribbons were fabricated from boehmite gels, followed by different heat treatments. The reinforcing effect of these carbon nanostructures was evidenced by compression tests carried out on the different alumina structures. A maximum relative increase of 50% in compressive strength was achieved for the γ-Al_2_O_3_ composite structure reinforced with reduced graphene oxide ribbons, which was also accompanied by an increase in the specific surface area.

## 1. Introduction

Boehmite, an aluminum oxide hydroxide (γ-AlO(OH)), is a well-known precursor of the family of transition aluminas, a series of crystalline intermediate phases obtained through thermal treatment that precede the final stable α-alumina (Al_2_O_3_) form [1]. Alumina-based materials are the most widely used advanced ceramics; hence, boehmite has been the subject of extensive studies and is increasingly relevant not only for its traditional use in the production of γ-Al_2_O_3_, the most important industrial catalyst support [2,3], but also for the fabrication of fine α-Al_2_O_3_ industrial components—refractories, electronic substrates, rotors, pumps or bearings [4,5]—and novel biomaterials [6].

One of the most recent and promising uses of boehmite is for the 3D printing of ceramics. When small amounts of certain acids are added to boehmite, the peptization process induces the formation of stable colloidal dispersions, allowing its application as a binder and plasticizer in the extrusion of α-Al_2_O_3_ powders through the control of pH, and mixing and drying steps [7]. The gel-forming ability of boehmite has brought attention to the study of boehmite hydrogels as suitable inks for direct ink writing (DIW), either as a precursor to monolithic Al_2_O_3_ structures or as a matrix for the development of composite scaffolds, utilizing its gelification characteristics to achieve printable inks without the further need for other additives (i.e., viscosifier or polyelectrolytes) for ink conditioning [8,9]. M’Barki et al. [10] indicated that gelation behavior is produced by successive hydrolysis and condensation reactions, leading to the partial dissolution of boehmite particles’ surface in monomeric aluminum species Al(H_2_O)_6_^3+^, and developed printable inks with a high content of solids (45 wt.%) utilizing a boehmite suspension with additions of nitric acid and α-Al_2_O_3_ seeds.

More recently, Zhang et al. [11] obtained highly porous 3D alumina structures with very high specific surface area (S_BET_) by foaming boehmite slurries. Likewise, Zheng et al. [12] manufactured porous structures by stabilizing oil-in-water emulsions with boehmite at 20 wt.%.

Porosity, as in the previous examples, is regulated by the intended application and often makes the structures more fragile. This characteristic, alongside some problems inherent in 3D printing technologies related to densification, impedes achieving good mechanical strength. Regarding this latter issue, the use of polymer composites printed by DIW and fused filament modeling (FDM), with fiber reinforcements in polymer ink or filament has shown, similar to bulk materials, to be an attractive alternative to obtain stronger structures [13,14]. In the case of 3D-printed ceramics, the use of fibers is beginning to be explored, displaying significant results in improving the flexural and compressive strength of the scaffolds. Some examples that demonstrate this mechanical enhancement are the manufacturing of reinforced SiC/carbon fiber [15] and tricalcium phosphate/boron nitride nanotubes [16] composites by selective laser sintering, the fabrication of aligned carbon fibers into silica composites by stereolithography [17], and zeolite/halloysite nanotubes structures obtained by DIW [18].

Carbon nanostructures—carbon nanotubes, graphene, fullerenes, ribbons and nanofibers—have been used as successful structural reinforcements while producing interesting functional modifications to polymer, ceramic, and metallic composites [19,20]. Notably, although the number of reported examples is still small, they also appear promising for the reinforcement of 3D-printed structures due to their small size and excellent properties. For instance, a content as low as 0.75 wt.% of carbon nanotubes (CNTs) was able to enhance the compressive yield strength of a hydroxyapatite/polycaprolactone composite by 55% [21]; Zhong et al. [22] also fabricated 3D-printed geopolymer/graphene oxide (GO) composites, noticing the enhancement of the printing characteristics produced by GO and encapsulation of geopolymer particles. The compressive strength of the structures increased by 260% by augmenting the GO content from 4 to 10 wt.%.

Considering the wide range of applications of alumina-based materials, as well as the possibilities of boehmite in the field of DIW, the aim of this work was to investigate the use of fiber-like carbon based nanostructures as reinforcements for reducing the brittleness of 3D-printed boehmite-derived Al_2_O_3_ structures. For this purpose, we fabricated 3D-printed composite structures of boehmite with a low content of CNTs or reduced GO ribbons, which were subsequently transformed into γ- and α-Al_2_O_3_ composites. The reinforcing capabilities of the carbon nanostructures for the two forms of aluminum oxide porous structures were studied by measuring the compressive response of the distinct scaffolds. To the best of our knowledge, this is the first time that GO ribbons were used in the reinforcement of 3D-printed engineering ceramics, and the results obtained, particularly for the light and brittle γ-Al_2_O_3_ phase, may serve as a guide for designing stronger porous materials with some added functionalities as well.

## 2. Materials and Methods

### 2.1. Materials Processing

Highly dispersible, surface-treated γ-AlO(OH) commercial powders (Dispal 11N7-80, Sasol, TX, USA, 220 nm dispersed particle size, 30 nm crystallite size, modified with 0.1 wt.% nitric acid) were used to fabricate the boehmite ink in Milli-Q water with a solids final content of 52 wt.%, assisted by ultrasonication and blade mixing, and later homogenized in a planetary centrifugal mixer (AR-250, Thinky Company, Laguna Hills, CA, USA) at 2000 rpm for 2 min.

GO ribbons were prepared by oxidation unzipping of CVD synthesized multiwall CNTs (MWCNT, ~700 µm length, Փ 160 nm), as described in a previous work [23], and reduced in ascorbic acid (A.A onwards) [24] to limit their hydrophilic nature and the number of potential anchoring sites for boehmite particles, thus minimizing the possibility of strong agglomeration. Reduced GO ribbons (rGO ribbons) were finally freeze-dried, sieved, and added to the recipient with the boehmite ink. The composite ink was homogenized in the planetary mixer at 2000 rpm for 2 min.

The same type of nanotubes used as a source for the ribbons was used for the composites with MWCNT [23]. To ensure homogenous dispersion and effective breaking of agglomerates, the nanotubes were mixed with boehmite powder by ball milling for 2 h using nylon balls. The powder mixture was sieved, added to a boehmite solution at 41 wt.%, and homogenized using the same parameters as for the boehmite/rGO ribbon ink. The content of carbon nanostructures, for both boehmite/MWCNT and boehmite/rGO ribbon composites, was set at 2 wt.% with respect to the solids in the ink. This relatively low content of the reinforcing fillers was chosen to avoid the possibility of agglomeration of the nanostructures.

The 3D structures were printed by DIW with a robocasting system (A3200, 3-D Inks LLC, Tulsa, OK, USA), loading the ink in a 3 cc printing syringe with a nozzle of 330 µm diameter (Precision Tips, EFD Inc., Westlake, OH, USA). Structures of 24 layers with dimensions of 7 × 7 × 6 mm^3^ were fabricated by orthogonal infill in adjacent layers with a rod separation of 0.8 mm, without a perimeter wall.

The scaffolds were air-dried for 24 h and thermally treated at maximum temperatures of 500 °C for 2 h and 1300 °C for 5 min to achieve the transformation of boehmite into γ- and α-Al_2_O_3_, respectively. The heat treatments were correspondingly carried out in a tubular furnace and a pressureless spark plasma sintering furnace (SPS, SPS-1050-CE, Fuji Electronic Industrial Co., Ltd., Tsurugashima, Saitama, Japan) in an inert atmosphere to avoid degradation of the carbon nanostructures.

### 2.2. Characterization

Dimensions and shrinkage of 3D structures after thermal treatments were determined using optical microscopes. Microstructure, fracture surface, and the external surface of the rods were observed by scanning electron microscopy (FE-SEM Hitachi S-4700 and SEM Hitachi TM-1000, Japan). The density and open porosity of the sintered samples were calculated by geometrical parameters using the method described in [25], taking 3.03, 3.65, 3.96, 1.90, and 2.10 g·cm^−1^ as the theoretical densities of boehmite, γ-Al_2_O_3_, α-Al_2_O_3_, MWCNT, and rGO ribbons, respectively.

Thermogravimetric analysis (Netzch STA 409/C, Selb, Germany) of the starting powders was carried out in an inert atmosphere to observe dehydration behavior from room temperature to 1000 °C using a heating rate of 5 °C·min^−1^. The transformation into Al_2_O_3_ phases was confirmed by X-ray diffraction (Bruker, D8 advance) of ground 3D structures. S_BET_ of the 3D structures was measured by N_2_ adsorption (Monosorb Analyser MS13, Quantachrome, FL, USA) with an error <0.5%, degassing the samples at a lower temperature than that at which they were treated. Raman spectroscopy (WITec Alpha300, Ulm, Germany) at 532 nm excitation wavelength was utilized to examine the reduction of GO ribbons, and maps of 20 × 20 µm^2^ were employed to detect the alignment of the fibers along the rods due to shear forces developed during printing.

The compressive response of the structures was investigated using a universal testing machine (ZwickiLine Z5.0 TS, Zwick-Roell, Ulm, Germany) with crosshead displacement of 0.5 mm·min^−1^, testing at least five specimens per material. The test was stopped after observing an abrupt load drop associated with cracking after the crushing point. The apparent elastic modulus was obtained from the slope of stress–strain curves in the linear region. The size of the test specimens was similar to the as-printed condition, except for the α-Al_2_O_3_ samples that were smaller (due to additional shrinkage during the high temperature treatment) but maintained the same geometry.

## 3. Results and Discussion

### 3.1. Boehmite and Al_2_O_3_ Structures

**Figure 1:**
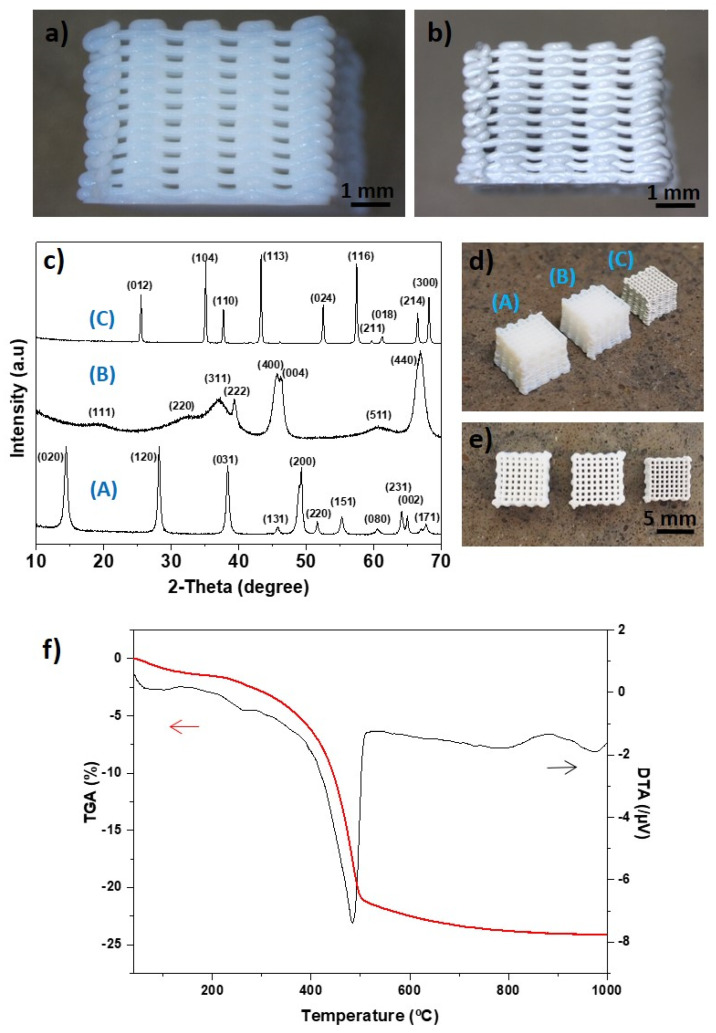
(**a**) Boehmite and (**b**) α-Al_2_O_3_ 3D-printed structures. The flattening of the bottom layers due to structure weight can be observed. (**c**) XRD patterns of the AlO(OH) structures (A) after printing, (B) treated at 500 °C for the transformation into the γ-Al_2_O_3_ phase, and (C) treated at 1300 °C for the transformation into the α-Al_2_O_3_ phase. (**d**,**e**) Side and top views of the structures placed in the same order showing significant shrinkage of the α-Al_2_O_3_ scaffold. (**f**) TGA-DTA of boehmite powder in an inert atmosphere.

The maximum boehmite content for the inks was 52 wt.%, as further loading produced a rapid hardening of the gel, making the homogenizing step in the planetary mixer difficult. For this content, good printability and shape retention were observed, with some flattening mainly at the bottom layers, produced by the weight of the structure during drying (Figure 1a,b). The aspect of the structures was translucent, becoming opaque as it increased in density and grain size upon treatment at 1300 °C. Figure 1c (A) shows the X-ray diffractogram of the as-printed boehmite structure, displaying sharp peaks characteristic of high crystallinity. TG-DTA produced a maximum endotherm peak at 486 °C (Figure 1f) related to the transformation into γ-Al_2_O_3_ [26]. Based on this result, the temperature selected for the first dehydration treatment was 500 °C. This crystalline form is reportedly stable in the range of 350–700 °C, but between 600 and 700 °C, transformation into the δ-Al_2_O_3_ phase could start [27,28]. The temperature of 1300 °C was selected for obtaining the α-Al_2_O_3_ phase, also above the reported transformation peak temperature for this phase, in the range of 1230–1270 °C [29]. The presence of these phases after dehydration treatments was confirmed, as it can be observed in the X-ray diffractograms (B) and (C) of Figure 1c, where the broad peaks and split (400) reflection in the γ-Al_2_O_3_ pattern are associated with structural disorder [28]. 

Samples treated at 500 °C presented an average weight loss of 14% that corresponded to adsorbed and structural water. Boehmite particles trap water on crystallite surfaces and are linked by hydrogen bonds; it is proposed that under heating at 500–600 °C during transformation into γ-Al_2_O_3_, hydrogen is transferred to hydroxyl groups, leading to the temporary formation of water [28]. This condensation reaction moves from the surface to internal layers and γ-Al_2_O_3_ still retains some hydroxyl groups that would be lost at higher temperatures [26], which is consistent with ~4 wt.% loss observed after treatment at 1300 °C and with the weight loss measured by TGA. Table 1 summarizes important properties of the boehmite-based structures for each treatment. The boehmite and γ-Al_2_O_3_ scaffolds were quite similar in size and overall appearance, as seen in Figure 1d,e; whereas the treatment at 1300 °C produced an important volumetric change (41% shrinkage). The γ-phase material reached a specific surface area of 126 m^2^·g^−1^, in agreement with values reported for γ-Al_2_O_3_ materials obtained from boehmite dehydration [26,30], although this property is greatly determined by the synthesis route of boehmite powders [26]. The crystallization of α-Al_2_O_3_ conveys an expected reduction in the specific surface area (9 m^2^·g^−1^).

**Figure 2:**
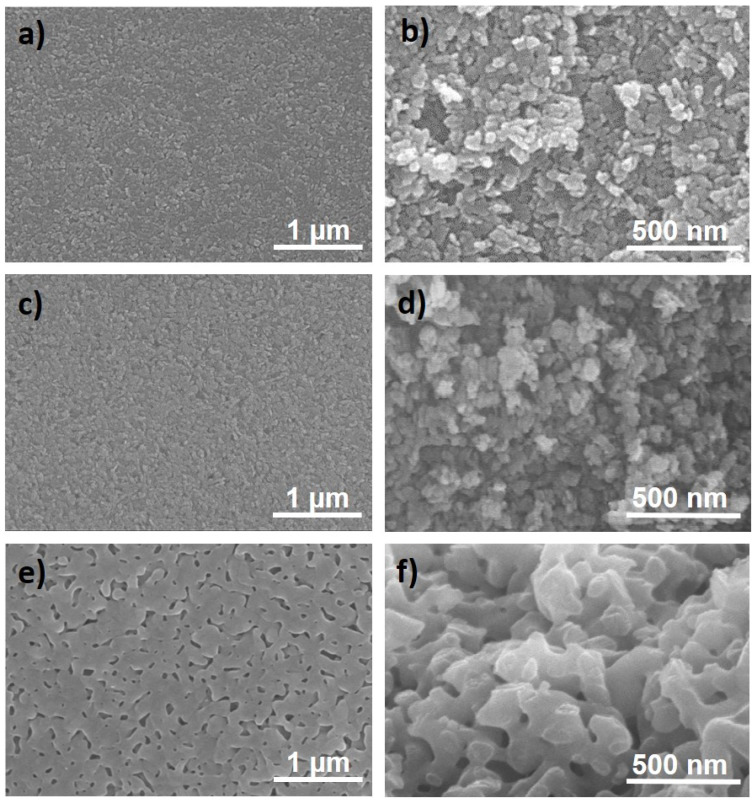
FESEM micrographs of rod surface (left column) and rod fracture surface (right column) in (**a**,**b**) bohemite, (**c**,**d**) γ-Al_2_O_3,_ and (**e**,**f**) α-Al_2_O_3_ scaffolds.

The microstructure of the different scaffolds is presented in Figure 2. As seen in Table 1, the structures are highly porous. Both rod surface and fracture surface are smooth in boehmite and γ-Al_2_O_3_ structures as a consequence of nanosized grains and pores. However, for α-Al_2_O_3_ material, noticeable grain growth and grain neck formation produced rough surfaces with bigger pores, in the order of 100–200 nm. Here, it is important to highlight that, despite the low density of structures, the rapid heating rate to 1300 °C of the SPS treatment without applied pressure was enough to achieve α-Al_2_O_3_ phase transformation due to the high effectiveness of the sintering technique. Dense alumina, sintered by the same technique and departing from boehmite powders, was reported at 1450 [31] or 1600 °C [32] and, in the case of 3D structures, they were sintered by conventional heating at 1300 °C for 2 h [11] or at 1300 °C for 1 h with α-Al_2_O_3_ seeded powders [10]. Such reduction in sintering time and temperature is of great use for protecting carbon nanostructures from thermal degradation/reaction in the composite scaffolds (as for bulk alumina/carbon nanostructure composites).

### 3.2. Composites with Carbon Nanostructures and their Reinforcing Capabilities 

**Figure 3:**
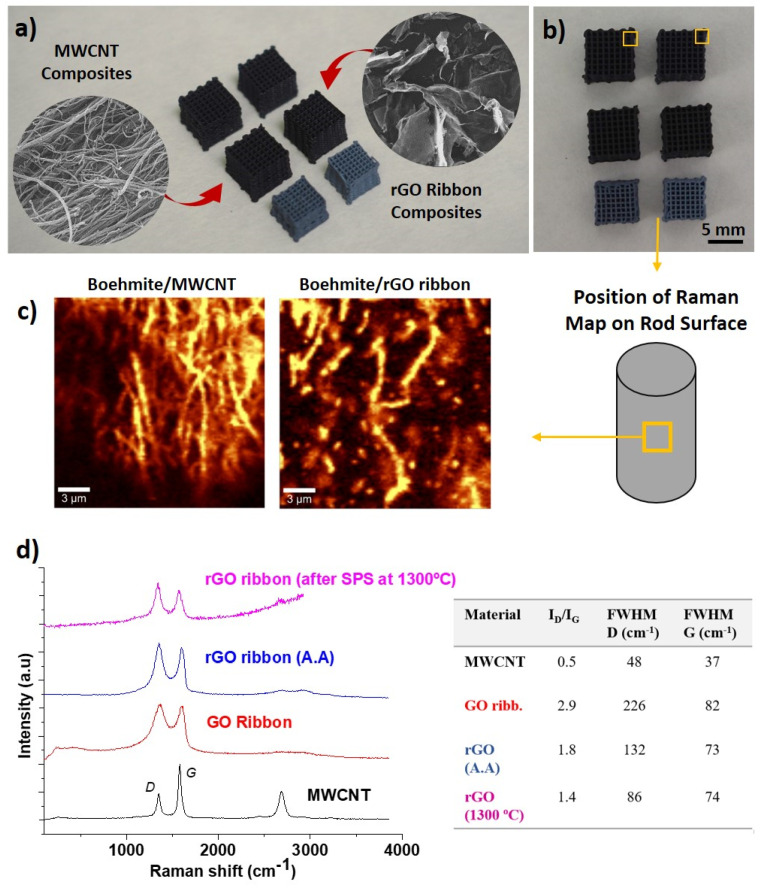
(**a**,**b**) Images of the composite scaffolds, arranged from top to bottom as follows: boehmite/MWCNT and boehmite/rGO ribbon; γ-Al_2_O_3_/MWCNT and γ-Al_2_O_3_/rGO ribbon; α-Al_2_O_3_/MWCNT and α-Al_2_O_3_/rGO ribbon. FESEM images of carbon nanostructures are inserted in (**a**). (**c**) Raman maps representing intensity of the carbon nanostructure’s G peak in an area of rod surface of boehmite/MWCNT and boehmite/rGO ribbon. (**d**) Raman spectra of rGO ribbons at different stages of synthesis and composite structures treatment. rGO (A.A) corresponds to the ascorbic-acid-treated GO ribbons. The table includes I_D_/I_G,_ the ratio of integrated areas of D and G peaks, and the full width at half maximum (FWHM) of the bands.

The substitution of 2 wt.% of boehmite by the lighter carbon nanostructures may affect the ink consistency as MWCNT and rGO ribbons exhibit highly hydrophobic and hydrophilic natures, respectively. However, no significant effects on the required shear thinning behavior were measured for ceramic inks containing either carbon nanostructures or graphene nanoplatelets [25,33]. The present inks were allowed to age for one week in a sealed container. This allowed the complete integration of the carbon nanostructures into the gel structure, which requires time. As we observed no segregation in the inks and printability was assured, we assumed that they assimilated quite homogeneously in the gel. Side and top views of the structures are shown in Figure 3a,b. Both MWCNT and ribbons composite scaffolds acquired the characteristic dark color of containing carbon materials, turning greyish with the transformation of the matrix into α-Al_2_O_3_. Total water loss in the scaffolds, calculated after treatment at 1300 °C, was in the range observed for pure boehmite samples.

The boehmite composites were studied by Raman spectroscopy mapping on the rod surface, visualizing the preferential alignment of the carbon nanostructures along the printing direction on the surface. The acquired maps, constructed by integration of MWCNT and rGO ribbons G peak, are presented in Figure 3c, where the brightest pixels correspond to nanostructures on the surface. The alignment is induced by shear forces that radially increase up to the wall of the syringe during printing, as observed in structures with fiber and platelet reinforcements. However, some differences in the alignment habit of nanotubes and ribbons exist due to the effects of the nanostructure’s size and shape. 1-D MWCNT look longer than 2-D rGO ribbons, which exhibit a large number of defects by the synthesis route (see high I_D_/I_G_ of rGO ribbons in Figure 3d) and could be shortened during the homogenizing step or adhere strongly in between boehmite particles. In addition, shear forces do not elongate them in the same way as MWCNT, as rGO ribbons can be folded and twisted due to a higher flexibility of the GO plane. At the rod core, both nanostructures would also be oriented in other directions, following the observed behavior of nanotubes that surround ceramic particles creating a network [34]. The table accompanying Raman spectra in Figure 3d shows the gradual recovery of rGO crystallinity with α-Al_2_O_3_ phase transformation (1300 °C), although the defect ratio is still elevated; in contrast, MWCNT did not show an increase in the I_D_/I_G_ ratio after 1300 °C (0.4, not included in the graph). It can be expected that the I_D_/I_G_ ratio is the result of defects left by partial elimination of functional groups (carboxyl, hydroxyl, and epoxide) and from disordered carbon regions. However, the presence of OH and O bonds cannot be disregarded, considering that rGO ribbons have a similar structure to rGO and, therefore, an analogous behavior under thermal reduction. X-ray photoelectron spectroscopy studies of rGO performed by the authors in a previous work [35] revealed that around 13–19% of C–OH, C=O, and C-O bonds remained for rGO samples treated at 1500 °C and ceramic/rGO composites sintered at 1625 °C. These differences in the defect/functionalization of carbon nanostructure surfaces play an important role in the mechanical properties, as discussed below.

The specific surface area of γ-Al_2_O_3_ increased from 126 to 136 m^2^·g^−1^ with the introduction of the nanostructures (Table 2), which was also accompanied by a higher rod porosity for these composites. We also observed 89% and 22% increases in specific surface area for the α-Al_2_O_3_ phase containing MWCNT and rGO ribbons, respectively. This latter finding, along with the highest density achieved by rGO ribbons composite, indicated that some rGO ribbons loss probably ocurred due to the effect of the high sintering temperature. In addition, higher densities could be obtained with rGO ribbons as the attachment to boehmite particles and bi dimensionality (size and flexibility) could favor better packing than nanotubes. 

**Figure 4:**
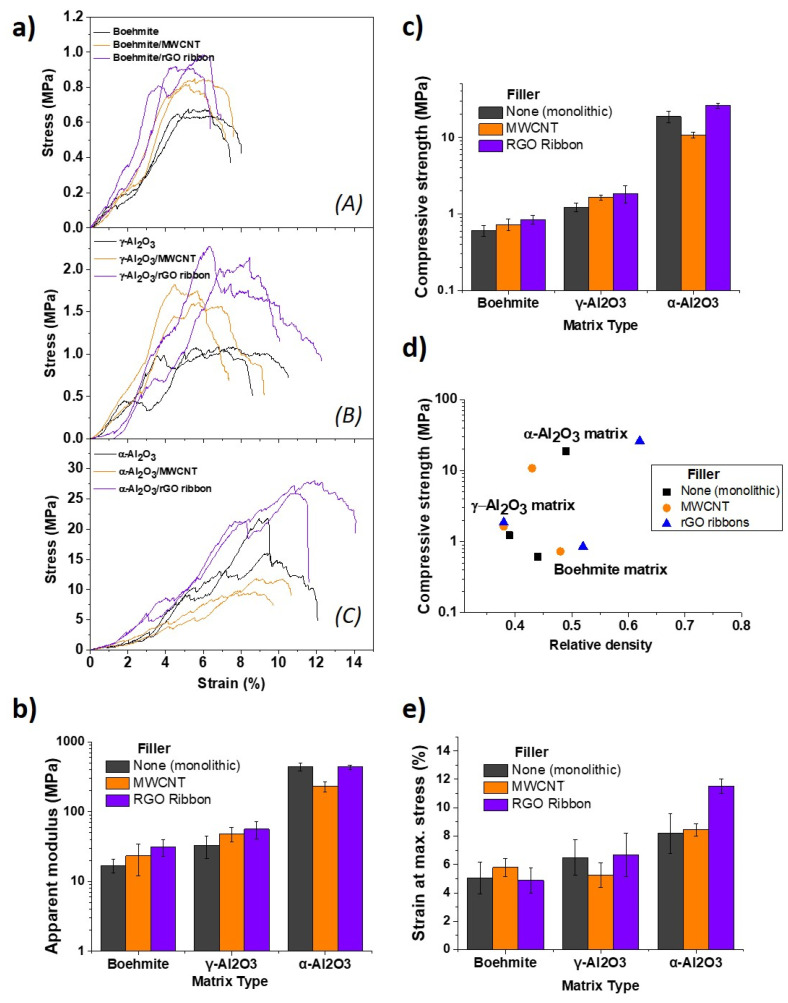
(**a**) Examples of stress–strain curves grouped by matrix material: (A) boehmite, (B) γ-Al_2_O_3_, and (C) α-Al_2_O_3_; (**b**) apparent modulus, (**c**) compressive strength, (**d**) compressive strength and density, and (**e**) strain at maximum stress.

Figure 4 shows the compressive response of the structures and Figure 4a presents examples of the stress–strain curves (two by material). The results for apparent modulus, compressive strength, and strain at maximum stress are summarized in Figure 4b,c,e histograms. As we are comparing structures with different densities, compressive strength is also represented with the relative density of each material (Figure 4d). The low resistance of the boehmite matrix is associated with weak hydrogen bonds between crystallites; increasing afterward during the transformation into γ-Al_2_O_3_, which produces the collapse of the layered boehmite lattice into spinel like crystals with some vacancies in the cation place. Finally, α-Al_2_O_3_ strong covalent bonding and grain neck formation lead to another increase in one order of magnitude in the compressive strength of the matrix. Strength values for the plain γ- and α-Al_2_O_3_ scaffolds are similar to the data reported for other porous structures based on alike materials, being influenced by the porosity and cell geometry. For instance: the elastic modulus of alumina aerogels showed values in the range of 1–100 MPa [36]; γ-Al_2_O_3_ samples sintered at 900 °C with 82% porosity presented compressive strength of 7.9 MPa [37]; porous α-Al_2_O_3_ structures sintered at 1400 °C displayed a compressive strength of 4 MPa [38]; and, for composite structures of alumina/feldspar sintered at 1200 °C with 70% porosity, a value of 6.80 MPa was reported [39].

Modulating (dividing) the maximum compressive stress by the density of the corresponding material, the largest reinforcing effects were obtained for the boehmite and γ-Al_2_O_3_ phases. These increases were 11% and 20% for MWCNT and rGO ribbons, respectively, for the boehmite matrix and 28% and 50% for the MWCNT and rGO ribbons in the case of the γ-Al_2_O_3_ matrix, respectively; finally, only nanoribbbons increased the compressive strength of the α-Al_2_O_3_ phase by 14%. This increase in the compressive resistances must be linked to a higher toughness as the defect size is larger in the composites, with the carbon nanostructures hindering multiple crack propagation during compression but being affected by the degree of interfacial bonding with each matrix. In the case of α-Al_2_O_3_, the presence of rGO ribbons could also help to reinforce the matrix against flaw nucleation produced by differential shrinkages.

**Figure 5:**
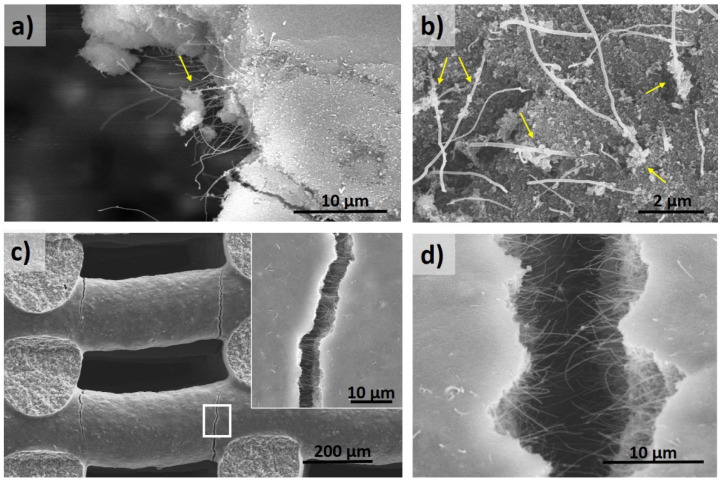
Micrographs of MWCNT composite structures after compression tests. (**a**,**b**) Pulled-out CNTs in boehmite/MWCNT composite showing boehmite particles adhered to some parts of the nanotubes. (**c**) Example of pulled-out nanotubes at cracks near α-Al_2_O_3_/MWCNT structure nodes, with nanotubes aligned in the direction parallel to the rod. (**d**) Close image of nanotubes in the Al_2_O_3_/MWCNT structure. Different from (**a**), the α-Al_2_O_3_ grains are not adhered to the nanotubes.

**Figure 6:**
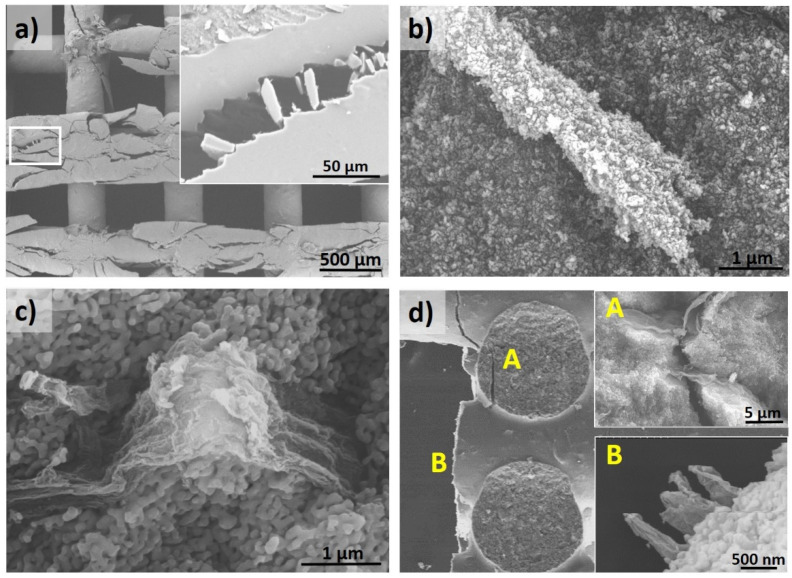
SEM images of fractured rGO ribbon composite structures after compression testing. (**a**) Crushed top layer of a boehmite/rGO ribbon structure showing varied damage. The inset shows a zoomed area in which pulled-out ribbons are completely covered by boehmite. (**b**) rGO ribbon protruding from the rod fracture surface in a γ-Al_2_O_3_/rGO ribbon structure. The γ-Al_2_O_3_ grains decorate the whole ribbon surface. (**c**) Rod fracture surface in an α-Al_2_O_3_/rGO ribbon structure. Ribbon morphology constituted by the graphene layers of an open nanotube is clearly appreciated. (**d**) Side view of a fractured α-Al_2_O_3_/rGO ribbon structure, showing examples of a bridged crack crossing structure node (A) and typical ribbon pull-out length observed at the edge (B).

The collapse of structures is caused by rod fracture, induced by the stresses accumulated at the edge of two rods’ joint, which are the weakest points. The inspection of samples after compression testing provided more insight into the mechanical response of the structures. For both MWCNT (Figure 5a,b) and rGO ribbons (Figure 6a,b) composites, the occurrence of boehmite and γ-Al_2_O_3_ particles adhering to pulled-out fillers was confirmed. This is an indication of the strong adhesion of the nanostructures to the matrix caused by the effect of hydroxyl functional groups in the matrix, which act as attaching sites. 

During initial loading, some areas of the boehmite/rGO ribbon composites could resemble brick-mortar behavior [40], with the ribbon phase allowing for strain release as ceramic zones remain intact. Moreover, as rGO ribbons present a functionalized surface also containing hydroxyl groups, the probable (weak) bonding effect with hydroxyl groups of boehmite and γ-Al_2_O_3_ may induce some friction during pull-out, which seems beneficial for reinforcement purposes; therefore, achieving the maximum relative increase in compressive strength. In the case of the α-Al_2_O_3_ matrix, the picture is very different due to microstructure and crystalline changes. MWCNT are no longer anchored to alumina grains (Figure 5d); the contact is reduced due to the porosity and dimensionality of MWCNT being easily pulled-out, as seen in the image. rGO ribbons are more easily bent and crumpled by the shrinkage of embedded alumina grains (Figure 6c,d) and hold a key defective surface, which keeps the load transfer enough to prevent crack propagation.

## 4. Conclusions

Porous γ-Al_2_O_3_ and α-Al_2_O_3_ scaffolds and their composites with fiber-like carbon nanostructures were successfully printed from boehmite inks. The treatment at 1300 °C by pressureless SPS was effective in achieving transformation into α-Al_2_O_3_. Carbon nanostructures appeared preferentially aligned due to shear forces during printing favoring reinforcement in the direction of the rod, but also in other directions due to their dimensionality and flexibility. The reinforcement produced by MWCNT and rGO ribbons in boehmite and γ-Al_2_O_3_ alumina matrices is strongly related to the interfacial bonding produced by the hydroxyl groups anchored to defective sites in MWCNT and functional groups in rGO ribbons. This condition changes with the structural transformation into the α-Al_2_O_3_ matrix, making MWCNT ineffective but not rGO ribbons, due to the bi-dimensional wrinkled structure and the remaining functional groups that are able to maintain clamped points for load transfer and, as such, result in a higher resistance to pull-out. This work sets a basis for achieving stronger 3D-printed porous materials with a low content of graphene and CNT fillers, which can simultaneously modify other properties such as specific surface area and electrical conductivity.

## Figures and Tables

**Table 1:** Density (ρ), porosity, and shrinkage of the monolithic boehmite and alumina structures.

3D-Structure	ρ (g·cm^−3^)	Total Porosity (%)	Rod Porosity (%)	Vol. Shrinkage (%)	Specific Surface Area (m^2^·g^−1^)
Boehmite	1.35	82	55	-	101
γ-Al_2_O_3_	1.43	85	60	9	126
α-Al_2_O_3_	1.95	80	50	41	9

**Table 2:** Density (ρ), porosity, and shrinkage of the composite structures.

3D-Structure	ρ (g·cm^−3^)	Total Porosity (%)	Rod Porosity (%)	Vol. Shrinkage (%)	Specific Surface Area (m^2^·g^−1^)
Boehmite/MWCNT	1.45	83	51	-	77
γ-Al_2_O_3_/MWCNT	1.36	87	62	2	136
α-Al_2_O_3_/MWCNT	1.67	84	57	34	17
Boehmite/rGO ribbon	1.58	81	47	-	83
γ-Al_2_O_3_/rGO ribbon	1.37	85	62	9	137
α-Al_2_O_3_/rGO ribbon	2.42	80	38	39	11

## Data Availability

Data are contained within the article.
